# Interactions between the Polysialylated Neural Cell Adhesion Molecule and the Transient Receptor Potential Canonical Channels 1, 4, and 5 Induce Entry of Ca^2+^ into Neurons

**DOI:** 10.3390/ijms231710027

**Published:** 2022-09-02

**Authors:** Laura Amores-Bonet, Ralf Kleene, Thomas Theis, Melitta Schachner

**Affiliations:** 1 Zentrum für Molekulare Neurobiologie, Universitätsklinikum Hamburg-Eppendorf, Falkenried 94, 20251 Hamburg, Germany; 2 Keck Center for Collaborative Neuroscience, Department of Cell Biology and Neuroscience, Rutgers University, 604 Allison Road, Piscataway, NJ 08854, USA

**Keywords:** NCAM, neural cell adhesion molecule, transient receptor potential canonical, ion channels, TRPC1, TRPC4, TRPC5, Ca^2+^ influx, polysialic acid, neurite outgrowth

## Abstract

The neural cell adhesion molecule (NCAM) plays important functional roles in the developing and mature nervous systems. Here, we show that the transient receptor potential canonical (TRPC) ion channels TRPC1, −4, and −5 not only interact with the intracellular domains of the transmembrane isoforms NCAM140 and NCAM180, but also with the glycan polysialic acid (PSA) covalently attached to the NCAM protein backbone. NCAM antibody treatment leads to the opening of TRPC1, −4, and −5 hetero- or homomers at the plasma membrane and to the influx of Ca^2+^ into cultured cortical neurons and CHO cells expressing NCAM, PSA, and TRPC1 and −4 or TRPC1 and −5. NCAM-stimulated Ca^2+^ entry was blocked by the TRPC inhibitor Pico145 or the bacterial PSA homolog colominic acid. NCAM-stimulated Ca^2+^ influx was detectable neither in NCAM-deficient cortical neurons nor in TRPC1/4- or TRPC1/5-expressing CHO cells that express NCAM, but not PSA. NCAM-induced neurite outgrowth was reduced by TRPC inhibitors and a function-blocking TRPC1 antibody. A characteristic signaling feature was that extracellular signal-regulated kinase 1/2 phosphorylation was also reduced by TRPC inhibitors. Our findings indicate that the interaction of NCAM with TRPC1, −4, and −5 contributes to the NCAM-stimulated and PSA-dependent Ca^2+^ entry into neurons thereby influencing essential neural functions.

## 1. Introduction

The two transmembrane NCAM isoforms NCAM140 and NCAM180 are generated by alternative splicing and play important roles as signal-transducing cell surface receptors in the nervous system [1,2]. These isoforms and their polysialylated variants (PSA-NCAM) mediate different, multifunctional signaling events leading to distinct cellular consequences in development, regeneration after injury, and synaptic plasticity and are associated with neuropsychiatric and neurodegenerative disorders [3,4,5,6,7,8,9,10,11].

Function-triggering NCAM antibodies or NCAM-derived peptides elicit several different signal transduction pathways, such as the activation of different protein kinase C (PKC) isoforms and phospholipase C (PLC) with subsequent generation of diacylglycerol (DAG) [12] and inositol trisphosphate (IP3), which enhance intracellular Ca^2+^ concentrations, resulting in cellular responses, such as neuritogenesis [1,13,14,15]. The NCAM-mediated promotion of neurite outgrowth depends also on the Ca^2+^-dependent binding of calmodulin to the intracellular domain (ICD) of NCAM140 and NCAM180 and on the NCAM-induced increase in cytoplasmic Ca^2+^ levels [16,17]. The inhibition of L- and N-type voltage-dependent Ca^2+^ channels does not completely block the NCAM-dependent elevation of cytoplasmic Ca^2+^ levels and the NCAM-induced neurite outgrowth [18], suggesting that other mechanisms or Ca^2+^ channels are involved in the recruitment of Ca^2+^ and stimulation of neurite outgrowth. Indeed, the TRPC inhibitor SKF96365 abolished the NCAM-induced increase in cytoplasmic Ca^2+^ levels and the promotion of neurite outgrowth [18], indicating that the NCAM-mediated elevation of the cytoplasmic Ca^2+^ level depends on TRPCs, of which the exact identity and mechanistic ways of function were not yet analyzed in detail.

The TRPC family comprises TRPC1, TRPC2, TRPC3, TRPC4, TRPC5, TRPC6, and TRPC7, which are nonselective cation channels permeable to Ca^2+^, sodium, and potassium ions [19,20,21,22,23,24,25,26,27,28,29,30,31,32,33,34,35,36,37,38,39,40,41,42,43]. The TRPCs are transmembrane proteins with six transmembrane segments, an intracellular N- and C-terminus, and three extracellular and two intracellular loops [44]. They can form homomers or heteromers [21] and function as receptor-operated or store-operated Ca^2+^ channels, which are activated by the PKC pathway as well as the production of DAG and IP3 [19,20,21]. In the case of receptor-operated Ca^2+^ entry, DAG directly activates TRPCs at the plasma membrane, while store-operated Ca^2+^ entry is triggered by the binding of IP3 to its receptor in endoplasmic reticulum (ER) membranes, leading to the release of Ca^2+^ into the cytoplasm and to increased Ca^2+^ levels, which then activate TRPCs in the plasma membrane.

TRPCs affect several neural functions, such as neuronal proliferation, survival, and differentiation as well as neurite outgrowth and axon guidance and, when impaired, are linked to diseases [19,23,33,40,41,42,45,46,47]. TRPCs and NCAM both interact with calmodulin, dopamine receptor D2, receptor tyrosine kinase TrkB, and fibroblast growth factor receptor [14,16,48,49,50,51,52,53]. Since NCAM and TRPCs share similar binding partners and functions, we hypothesized that NCAM interacts directly with TRPCs and thereby influences their functions. Here, we show that not only the NCAM180 and NCAM140 protein backbones, but also the covalently attached PSA interact with TRPC1, −4, and −5, but not with TRPC3, −6, or −7. The ICDs of the NCAM isoforms and the bacterial PSA homolog colominic acid (colominic acid/PSA) bind directly to the N-terminal ICDs of TRPC1, −4, and −5. We further show that stimulation of NCAM-dependent signaling pathways induces NCAM- and PSA-NCAM-dependent Ca^2+^ entry into cortical neurons via TRPC1, −4, and −5 homo- or heteromers, resulting in enhanced neurite outgrowth.

## 2. Results

### 2.1. NCAM140 and NCAM180 Are Associated with TRPC1, −4, and −5 in the Mouse Brain

**Figure 1:**
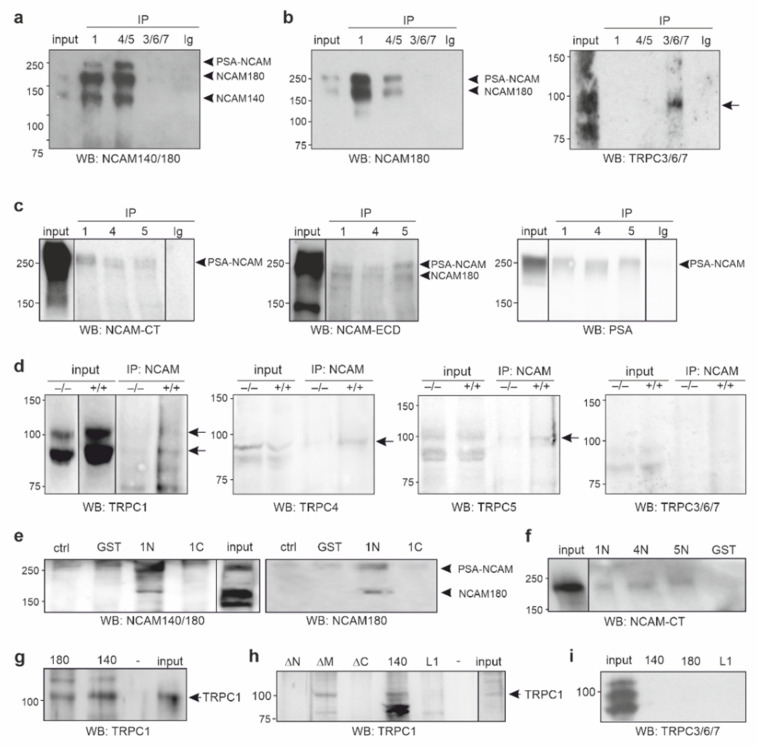
NCAM and TRPC1, −4, and −5 interact via their ICDs. (**a**,**b**) A brain detergent extract was used for immunoprecipitation (IP) with TRPC1 antibody H-105, TRPC4/5 antibody H-80 (4/5), TRPC3/6/7 antibody H-100 (3/6/7), and nonimmune control antibody (Ig). The brain detergent extract (input) and the immunoprecipitates were subjected to Western blot (WB) analysis with NCAM antibody 5B8 (NCAM140/180) (**a**), NCAM antibody D3 (NCAM180), or TRPC3/6/7 antibody H-100 (3/6/7) (**b**). (**c**) Detergent extracts of membrane-enriched brain fractions (input) and TRPC1 antibody ACC-010 (1), TRPC4 antibody ACC-018 (4), TRPC5 antibody ACC-020 (5), and a nonimmune control antibody (Ig) were used for immunoprecipitation, and the NCAM antibody GTX133217 against the C-terminus (NCAM-CT), a chicken antibody against the extracellular domains of NCAM (NCAM-ECD), and PSA antibody 735 (PSA) were used for Western blot analysis. (**a**–**c**) Arrowheads indicate bands representing NCAM140, NCAM180, and polysialylated NCAM140 and NCAM180 (PSA-NCAM), and the arrow indicates TRPC3, −6, or −7 bands. (**d**) Immunoprecipitation was carried out with brain detergent extracts (input) from wild-type (+/+) and NCAM-deficient (−/−) mice using NCAM antibody H28 and followed by Western blot analysis with TRPC1 antibody E-6 (1), TRPC4 antibody N77/15 (4), TRPC5 antibody N67/15 (5), and TRPC3/6/7 antibody H-100 (3/6/7). Arrows indicate TRPC1, −4, or −5 bands. (**e**,**f**) NCAM antibody 5B8 (NCAM140/180), D3 (NCAM180) (**e**), and GTX133217 (NCAM-CT) (**f**) were used for Western blot analysis of precipitates from pull-down experiments using detergent brain extracts (input) (**e**) or a membrane-enriched fraction (input) (**f**) and beads only (ctrl) or beads with GST, GST-tagged N-terminal (1N), or C-terminal (1C) ICDs of TRPC1 or GST-tagged N-terminal ICDs of TRPC4 (4N) or TRPC5 (5N). Arrowheads indicate NCAM180 and PSA-NCAM bands. (**g**–**i**) TRPC1 antibody E-6 (**g**,**h**) or TRPC3/6/7 antibody H-100 (**i**) were used for Western blot analysis of precipitates from pull-down experiments with detergent brain extracts and His-tagged ICDs of NCAM140 (140) (**g**–**i**), NCAM180 (180) (**g**,**i**), L1 (**h**,**i**), or NCAM140 with deletion of the N-terminal (ΔN), middle (ΔM), or C-terminal (ΔC) part (**h**). Arrows indicate TRPC1 bands.

NCAM stimulation elevates cytoplasmic Ca^2+^ levels, which are reduced by SKF96365, an inhibitor that affects all TRPCs [18]. Since TRPCs can function as store-operated and/or receptor-operated Ca^2+^ channels [19,20,24,25], we investigated which mechanism is triggered by NCAM stimulation. We first performed immunoprecipitations with detergent extracts from young adult mouse brains and antibodies against TRPC1, TRPC4 and −5 (TRPC4/5) or TRPC3, −6, and −7 (TRPC3/6/7). Western blot analysis with the NCAM antibody 5B8 recognizing both NCAM140 and NCAM180 showed three NCAM-positive bands of 140, 180, and 250 kDa in the TRPC1 and TRPC4/5 immunoprecipitates, while no NCAM-positive bands were seen in the TRPC3/6/7 immunoprecipitates or control immunoprecipitates with the nonimmune antibody (Figure 1a). In TRPC1 and TRPC4/5 immunoprecipitates, but not in the TRPC3/6/7 or control immunoprecipitates, the NCAM180-specific antibody D3 showed two bands of 180 and 250 kDa (Figure 1b) with the 250 kDa band representing PSA-NCAM. Bands for TRPC3, −6, or −7 were detected in the TRPC3/6/7 immunoprecipitate and input, but not in the TRPC1, TRPC4/5, or control immunoprecipitates (Figure 1b), showing that the TRPC3/6/7 antibody precipitated its antigens, but not NCAM. Using a detergent-solubilized membrane-enriched brain fraction and TRPC1, −4, and −5 antibodies for immunoprecipitation, antibodies against C-terminal NCAM epitopes, extracellular NCAM domains, or PSA recognized diffuse 250 kDa PSA-NCAM bands in the TRPC1, −4, and −5 immunoprecipitates, but not in the nonimmune control immunoprecipitates (Figure 1c).

Using a brain detergent extract and an antibody against the extracellular NCAM domains for immunoprecipitation, TRPC1-, TRPC4-, and TRPC5-positive bands were detected by TRPC1, −4, and −5 antibodies in the NCAM immunoprecipitates from extracts of wild-type brains, while these bands were hardly detectable in NCAM immunoprecipitates from extracts of NCAM-deficient brains (Figure 1d). No bands were detected in NCAM immunoprecipitates when the TRPC3/6/7 antibody was used for Western blot analysis (Figure 1d). These results indicate that NCAM140 and NCAM180 with or without PSA associate with TRPC1, −4, and −5.

### 2.2. The ICDs of NCAM140 and NCAM180 Interact with the N-Terminal ICDs of TRPC1, −4, and −5

To analyze whether the N- or C-terminal ICD of TRPC1 mediates the interaction with NCAM, we performed pull-down experiments with brain extracts and recombinant N- and C-terminal TRPC1-ICDs, which carry a glutathione S-transferase (GST) tag. Western blot analysis with NCAM antibodies 5B8 and D3 showed that the GST-tagged N-terminal TRPC1-ICD, but neither the GST-tagged C-terminal TRPC1-ICD nor GST alone, precipitated NCAM180 and PSA-NCAM at 250 kDa, but only very small amounts of NCAM140 (Figure 1e). In a pull-down experiment using a detergent-solubilized membrane-enriched brain fraction and the GST-tagged N-terminal ICDs of TRPC1, −44 and −5, precipitation of PSA-NCAM by the GST-tagged N-terminal TRPC1-ICD, TRPC4-ICD, and TRPC5-ICD, but not GST, was observed (Figure 1f). The results indicate that mainly nonpolysialylated NCAM180 and polysialylated NCAM180 or NCAM140 associate with the N-terminal ICDs of TRPC1, −4, and −5.

In a reverse pull-down experiment, recombinant His-tagged NCAM180-ICD and NCAM140-ICD coprecipitated TRPC1 from brain extracts (Figure 1g), indicating that NCAM180 and NCAM140 interact with TRPC1. Using extracts and NCAM140-ICD lacking either amino acids 1–44 at the N-terminus, 29–66 in the middle part, or 76–120 at C-terminus for pull-down, NCAM140-ICD lacking the N- or C-terminal amino acids did not precipitate TRPC1, whereas NCAM140-ICD lacking the middle part precipitated TRPC1 (Figure 1h). L1-ICD did not precipitate TRPC1 (Figure 1h). These results indicate that amino acids at the N- and C-terminus of NCAM140-ICD are required for the interaction with TRPC1. Of note, recombinant His-tagged NCAM180-ICD, NCAM140-ICD, or L1-ICD did not coprecipitate TRPC3, −6, or −7 from brain extracts (Figure 1i), supporting the notion that neither NCAM180 nor NCAM140 interact with these TRPCs.

**Figure 2:**
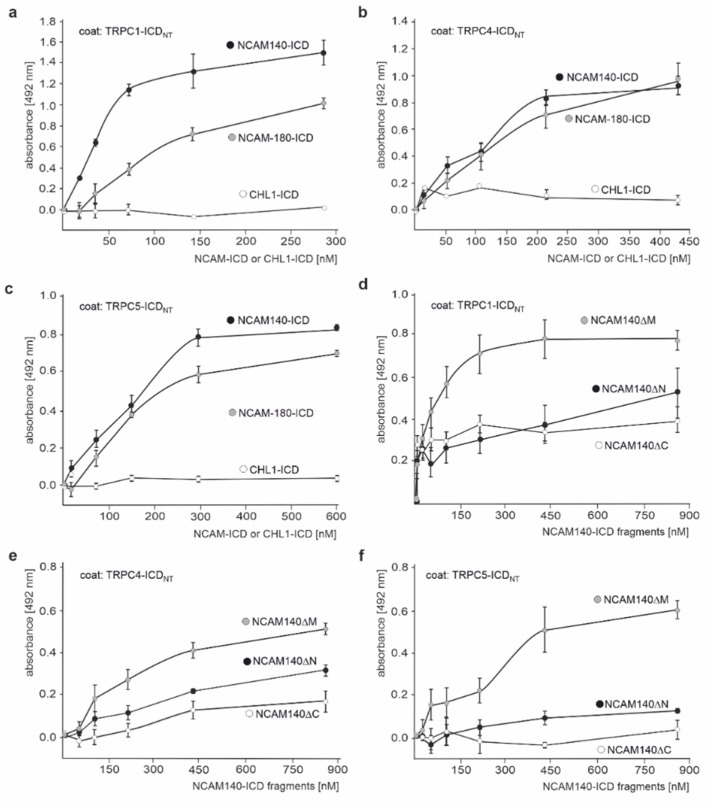
NCAM140-ICD and NCAM180-ICD directly bind to the N-terminal ICDs of TRPC1, −4, and −5. Recombinant N-terminal ICDs of TRPC1 (TRPC1-ICD_NT_) (**a**,**d**), TRPC4 (TRPC4-ICD_NT_) (**b**,**e**), and TRPC5 (TRPC5-ICD_NT_) (**c**,**f**) were substrate-coated and incubated with increasing concentrations of recombinant NCAM140-ICD (**a**–**c**), NCAM180-ICD (**a**–**c**), CHL1-ICD (**a**–**c**), or NCAM140-ICD with a deletion of the N-terminal (NCAM140ΔN), middle (NCAM140ΔM), or C-terminal (NCAM140ΔC) parts (**d**–**f**). Binding was determined by ELISA using NCAM antibody P61 (**a**–**c**), CHL1 antibody (**a**–**c**), and NCAM antibody GTX133217 (**d**–**f**). Mean values ± SEM from 3 independent experiments carried out in triplicates are shown.

To test whether TRPC-1, −4, and −5 directly bind to NCAM140 and NCAM180 via their ICDs, ELISA was performed with recombinant N-terminal ICDs of TRPC1, −4, or −5 and recombinant C-terminal ICDs of NCAM140 or NCAM180. Both NCAM140-ICD and NCAM180-ICD showed a concentration-dependent and saturable binding to TRPC1-, TRPC4-, and TRPC5-ICD, while the ICD of the close homolog of L1 (CHL1-ICD) did not bind (Figure 2a–c). ELISA with NCAM140-ICD lacking either the N-terminal, middle, or C-terminal part showed a concentration-dependent binding to TRPC1-, TRPC4-, and TRPC5-ICD only for NCAM140-ICD lacking the middle part (Figure 2d–f), indicating that amino acids at the N- and C-terminus interact with the N-terminal ICDs of TRPC1, −4, and −5.

**Figure 3:**
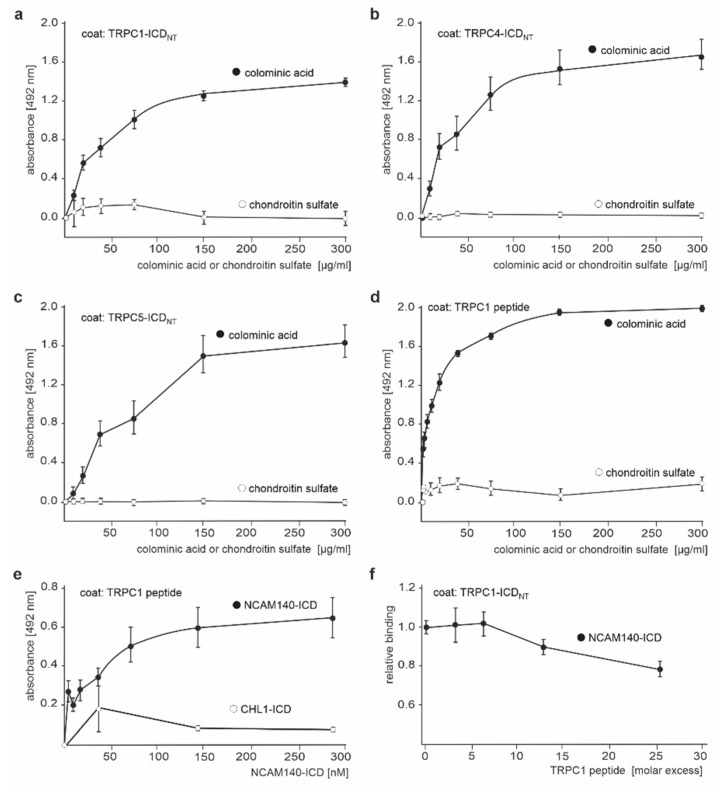
Colominic acid/PSA binds to the N-terminal ICDs of TRPC1, −4, and −5, and its binding site overlaps with the binding site of NCAM140-ICD. Recombinant N-terminal ICDs of TRPC1 (TRPC1-ICD_NT_) (**a**,**d**), TRPC4 (TRPC4-ICD_NT_) (**b**), TRPC5 (TRPC5-ICD_NT_) (**c**), and the TRPC1 peptide (**d**) were substrate-coated and incubated with increasing concentrations of colominic acid/PSA or chondroitin sulfate. Binding was determined by ELISA with antibodies against PSA or chondroitin sulfate. Mean values ± SEM from 3 independent experiments carried out in triplicates are shown. TRPC1 peptide (**e**) or N-terminal TRPC1-ICD (TRPC1-ICD_NT_) (**f**) were substrate-coated and incubated with increasing concentrations of NCAM140-ICD or CHL1-ICD (**e**) or with one NCAM140-ICD concentration and different concentrations of TRPC1 peptide (**f**). Binding was determined by ELISA using NCAM antibody P61 (**e**,**f**) and CHL1 antibody C-18 (**e**). Mean values ± SEM from 3 independent experiments carried out in triplicates are shown.

Since PSA at the cell surface binds to intracellular myristoylated alanine-rich C kinase substrate (MARCKS) within the plane of the plasma membrane [54], and since MARCKS was found to bind to TRPC1 [55,56], we tested whether PSA could also bind to the N-terminal TRPC-ICDs. ELISA showed a saturable and concentration-dependent binding of colominic acid/PSA to TRPC1-, TRPC4-, and TRPC5-ICD, while chondroitin sulfate did not bind (Figure 3a–c). PSA binds to an amphipathic helical structure in the effector domain of MARCKS [54], and a sequence analysis of TRPC1, −4, and −5 showed that the conserved sequence LARQCKMFAKDLLAQARN at positions 261–278 in the N-terminal ICD of murine TRPC1 and similar conserved sequence stretches in the N-terminal ICDs of TRPC−4 and −5 may form amphipathic helical structures. To test whether PSA binds to amphipathic helical structures in the N-terminal ICD of TRPC1, a synthetic TRPC1 peptide containing the putative amphipathic helical structure-forming sequence LARQCKMFAKDLLAQARN was used for ELISA with colominic acid/PSA. Colominic acid/PSA bound in a concentration-dependent manner to the substrate-coated TRPC1 peptide, while chondroitin sulfate did not bind (Figure 3d). NCAM140-ICD, but not CHL1-ICD, also bound to the substrate-coated TRPC1 peptide in a concentration-dependent manner, and the binding of NCAM140-ICD to TRPC1-ICD was reduced by the TRPC1 peptide in a competition ELISA (Figure 3e,f), indicating that the binding sites for NCAM and PSA in TRPC1-ICD overlap and suggesting that the binding of PSA to the amphipathic helical structures in the N-terminal ICDs of TRPC1, −4, and −5 interferes with NCAM binding to these TRPCs.

### 2.3. NCAM Colocalizes with TRPC1, −4, and −5 at the Neuronal Plasma Membrane

**Figure 4:**
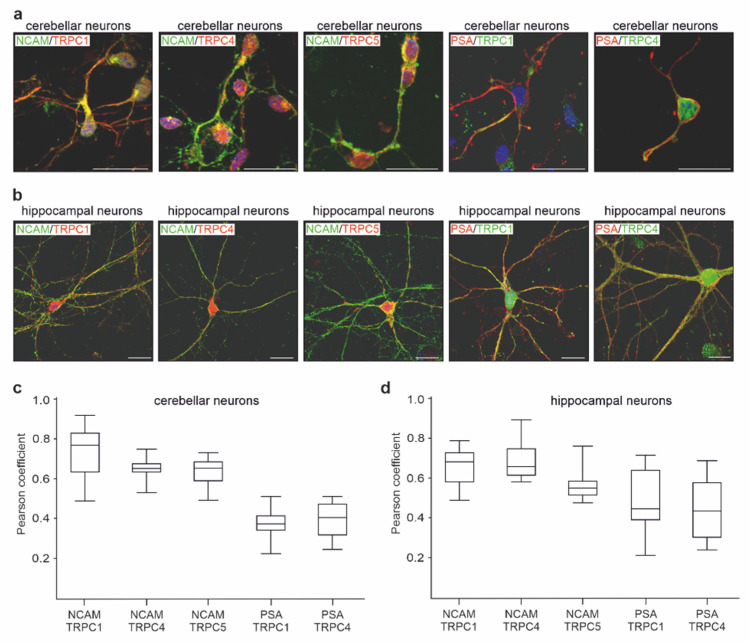
NCAM colocalizes with TRPC1, −4, and −5 in hippocampal and cerebellar neurons. Cultured neurons were subjected to double immunostaining with rabbit NCAM antibody GTX133217 and mouse TRPC1 antibody E-6, TRPC4 antibody N77/15, or TRPC5 antibody N67/15, as well as with mouse PSA antibody 735 and rabbit TRPC1 antibody GTX54876 or rabbit TRPC4 antibody ACC-119. Nuclei were stained with DAPI (blue). (**a**,**b**) Representative images are shown for coimmunostaining of TRPC1 (red) and NCAM (green) or of TRPC1 (green) and PSA (red) in cerebellar (**a**) and hippocampal (**b**) neurons. Superimposition of red and green stainings shows colocalization in yellow. Scale bar: 20 µm. (**c**,**d**) Pearson’s coefficients were determined from 10 immunostained neurons per group and from 2 different cultures. Box plots show the Pearson’s coefficients and indicate an overlap of NCAM or PSA stainings with TRPC stainings in cerebellar (**c**) and hippocampal (**d**) neurons.

**Figure 5:**
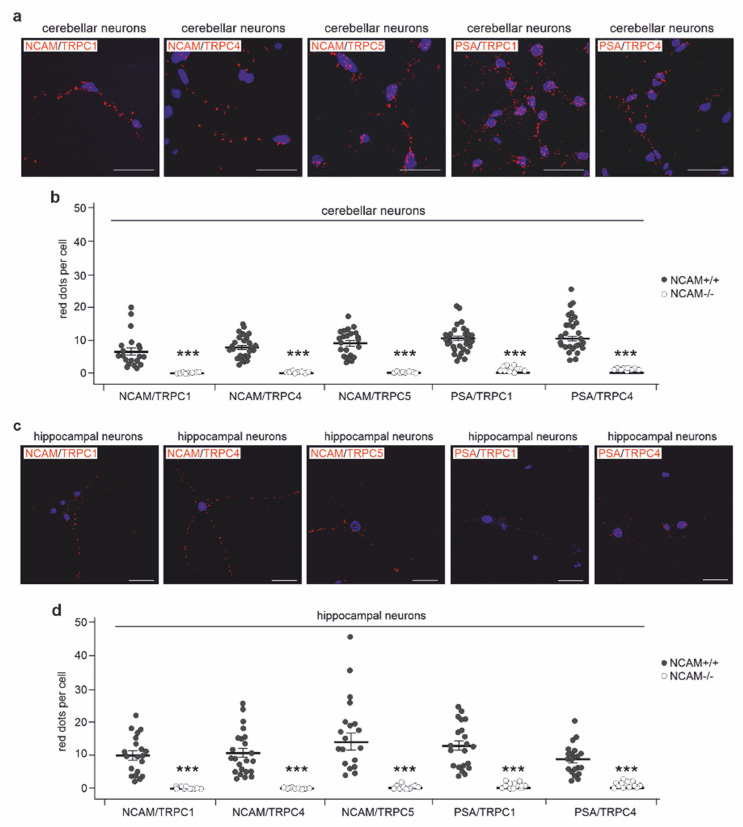
NCAM localizes in close proximity to TRPC1, −4, and −5 in hippocampal and cerebellar neurons. Cultured neurons from wild-type (NCAM+/+) and NCAM-deficient (NCAM−/−) mice were subjected to proximity ligation assay with rabbit NCAM antibody GTX133217 and mouse TRPC1 antibody E-6, TRPC4 antibody N77/15, or TRPC5 antibody N67/15, as well as with mouse PSA antibody 735 and rabbit TRPC1 antibody GTX54876 or TRPC4 antibody ACC-119. Nuclei were stained with DAPI (blue). Representative images are shown for TRPC1, −4, or −5 and NCAM or PSA in cerebellar (**a**) and hippocampal (**c**) neurons (scale bar: 20 µm). Red dots indicate close proximity of NCAM or PSA with TRPCs in cerebellar (**b**) and hippocampal (**d**) neurons. (**b**,**d**) Numbers of red dots were determined from 2 independent experiments analyzing 10 neurons per group and experiment. Scatter plots show mean values ± SEM and single values for the numbers of red dots per cell (*** *p* < 0.001 relative to wild-type neurons; Mann–Whitney test).

**Figure 6:**
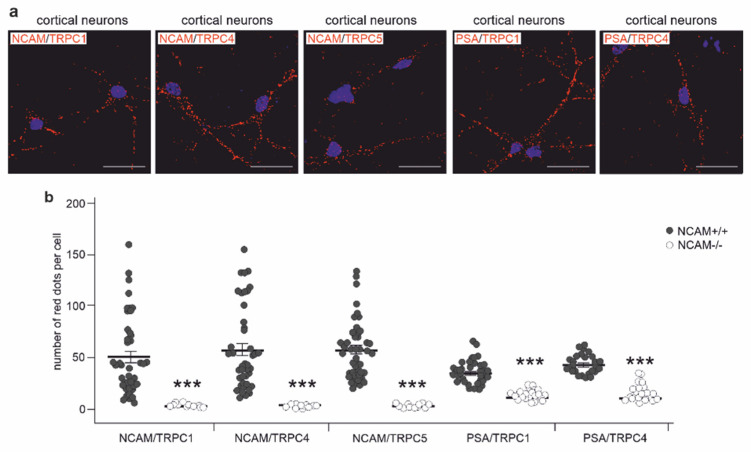
NCAM localizes in close proximity to TRPC1, −4, and −5 in cortical neurons. (**a**,**b**) Cultured cortical neurons from wild-type (NCAM+/+) and NCAM-deficient (NCAM−/−) mice were subjected to proximity ligation assay with rabbit NCAM antibody GTX133217 and mouse TRPC1 antibody E-6, TRPC4 antibody N77/15, or TRPC5 antibody N67/15, as well as with mouse PSA antibody 735 and rabbit TRPC1 antibody GTX54876 or TRPC4 antibody ACC-119. Nuclei were stained with DAPI (blue). (**a**) Representative images are shown for proximity ligation of TRPCs and NCAM or PSA in wild-type neurons (scale bar: 20 µm). Red dots indicate close proximity of NCAM or PSA with TRPCs in wild-type, but not NCAM-deficient neurons. Numbers of red dots from 3 independent experiments analyzing 10 neurons per group and experiment were determined. (**b**) Scatter plots show mean values ± SEM and single values for numbers of red dots per cell (*** *p* < 0.001 relative to wild-type neurons; Mann–Whitney test).

To investigate whether NCAM and PSA could interact with TRPC1, −4, and −5 in a cellular context, we first performed double immunostaining of hippocampal and cerebellar neurons with antibodies against NCAM and TRPC1, −4, or −5. Pearson’s analysis of images indicated a strong colocalization of NCAM with the TRPCs (Figure 4a–c). When the antibodies against PSA and the extracellular TRPC1 and −4 domains were used for immunostaining, Pearson’s analysis also showed a moderate colocalization of PSA with these TRPCs (Figure 4a–c). By proximity ligation that allows the detection of proteins, which are at a distance of less than 40 nm, NCAM/TRPC1-, NCAM/TRPC4-, NCAM/TRPC5-, PSA/TRPC1-, and PSA/TRPC4-positive red dots indicative for a close association were observed in hippocampal and cerebellar neurons from wild-type, but not NCAM-deficient mice (Figure 5a–d). Proximity ligation assays with cortical neurons from wild-type and NCAM-deficient mice showed high numbers of NCAM/TRPC1-, NCAM/TRPC4-, NCAM/TRPC5-, PSA/TRPC1-, and PSA/TRPC4-positive red dots in wild-type, but not NCAM-deficient neurons (Figure 6a,b). Immunostaining and proximity ligation with TRPC5 and PSA antibodies could not be performed, because appropriate antibodies against the extracellular domain of TRPC5 were not available.

**Figure 7:**
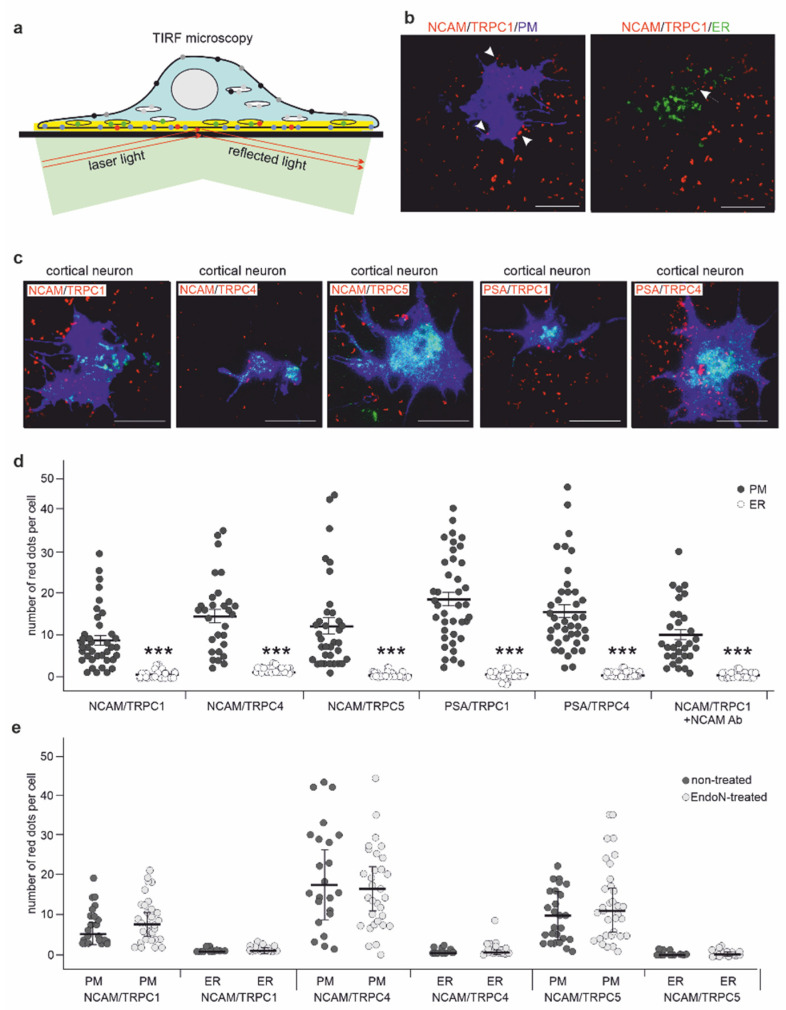
NCAM colocalizes with TRPC1, −4, and −5 predominantly in the plasma membrane of cortical neurons. (**a**) The scheme illustrates the principle of TIRF microscopy. A glass coverslip (black bar) and an adhering neuron (light blue) with nucleus and ER (ovals) are depicted. Application of laser light (light green; red arrows) at a certain angle leads to total reflection of light at the interfaces of the glass coverslip and the aqueous film between coverslip and adherent cells, generating an evanescent wave, which creates an evanescent field (yellow area). As the energy in this field decreases exponentially with distance of typically 60–100 nm to the interfaces, only fluorophores in this proximity to the coverslip are excited, leaving other fluorophores in the cell not excited. This allows the detection of protein interactions at the plasma membrane and in the ER membranes near the plasma membrane. Shown within the evanescent field are excited fluorophores indicating TRPC/NCAM interactions (red circles), plasma membrane (PM) marker proteins (blue circles), and ER membrane marker proteins (green circles). Outside of this field, light grey, dark grey, and black circles indicate the corresponding nonexcited fluorophores. (**b**–**e**) Cultured wild-type cortical neurons were first transfected with plasmids encoding a plasma membrane-associated fusion protein of blue fluorescence protein and the CAAX motif-containing sequence of H-Ras or encoding an ER membrane-associated fusion protein of enhanced green fluorescence protein, the ER-targeting sequence of calreticulin, and the ER retention sequence KDEL. Subsequently, the cells were treated without (**d**,**e**) or with the NCAM antibody (**d**) or EndoN (**e**) and subjected to proximity ligation with rabbit NCAM antibody GTX133217 and mouse TRPC1 antibody E-6, TRPC4 antibody N77/15, or TRPC5 antibody N67/15 or with mouse PSA antibody 735 and rabbit TRPC1 antibody ACC-010 or TRPC4 antibody ACC-018. The neurons were then analyzed by TIRF microscopy. (**b**) Representative images show TRPC1/NCAM interactions (red dots) at the cell surface predominantly near the plasma membrane marker protein (blue) (NCAM/TRPC1/PM), but rarely near the ER membrane marker protein (green) (NCAM/TRPC1/ER) (scale bar: 20 µm). (**c**) Representative images are shown for interactions of TRPCs with NCAM or PSA at the cell surface (scale bar: 20 µm). The numbers of red dots near to the plasma membrane marker (blue) or to the ER marker (green) were counted separately (overlapping dots were not considered) and indicate the interaction of NCAM or PSA with TRPCs in the plasma membrane or in ER membranes near the plasma membrane. (**d**,**e**) Scatter plots show mean values ± SEM and single values for the numbers of plasma membrane-associated (PM) and ER-associated (ER) red dots per cell from 3 independent experiments analyzing 10 neurons per group and experiment (*** *p* < 0.001; Mann–Whitney test comparing PM and ER values).

To determine whether NCAM colocalizes with TRPCs in the plasma membrane or ER membranes, cortical neurons were analyzed by total internal reflection fluorescence (TIRF) microscopy (Figure 7a). To distinguish between plasma membrane and ER localization, cortical neurons were transfected with plasmids encoding a plasma or ER membrane-associated protein. After transfection, neurons were fixed, detergent-permeabilized, and subjected to proximity ligation assay with NCAM, PSA, TRPC1, TRPC4, and TRPC5 antibodies and to TIRF analysis. High numbers of NCAM/TRPC1-, NCAM/TRPC4-, NCAM/TRPC5-, PSA/TRPC1-, and PSA/TRPC4-positive red dots were seen near the plasma membrane marker, while only a few red dots were detected near the ER marker (Figure 7b–d). Interestingly, treatment with a function-triggering NCAM antibody or removal of PSA by the PSA-degrading enzyme endoneuraminidase N (EndoN) [57] did not alter the numbers and localization of red dots (Figure 7d,e). The results indicate that PSA-NCAM is associated with the TRPC1, −4, and −5 at the plasma membrane and that stimulation with the NCAM antibody does not affect this association.

### 2.4. NCAM Regulates the Influx of Ca^2+^ via TRPC1, −4, and −5

**Figure 8:**
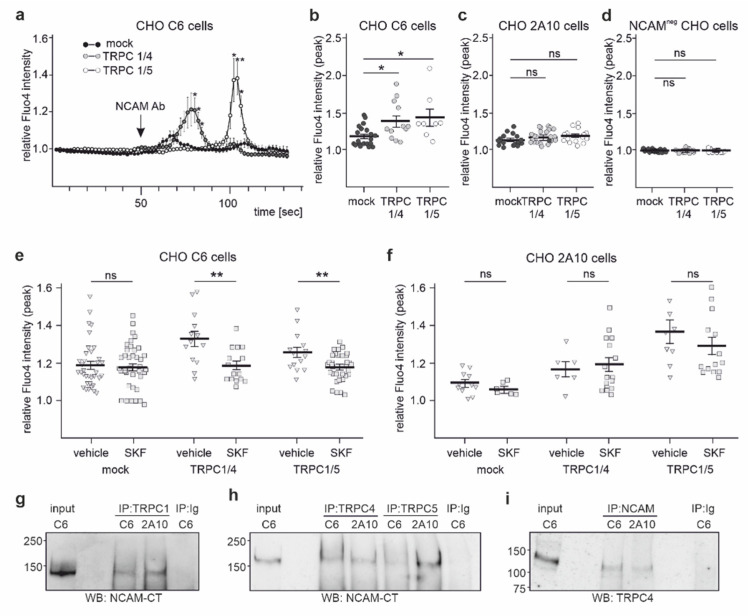
NCAM regulates the Ca^2+^ entry via TRPC1, −4, and −5 in transfected CHO cells that express PSA-NCAM. NCAM-expressing CHO 2A10 cells, PSA-NCAM-expressing CHO C6 cells, or NCAM-lacking NCAM^neg^ CHO cells were mock-transfected or transfected with plasmids encoding TRPC1/4 or TRPC1/5 heteromers, loaded with the Ca^2+^ indicator Fluo-4 AM, treated with or without function-triggering NCAM antibody in the absence or presence of the TRPC inhibitor SKF96365, and subjected to Ca^2+^ imaging. (**a**) A representative time course of a Ca^2+^ response of mock-transfected and TRPC1/4- or TRPC1/5-transfected CHO C6 cells after application of a function-triggering NCAM antibody (arrow; NCAM Ab) is shown (* *p* < 0.05, ** *p* < 0.01; mixed effects analysis comparing values from the TRPC1/4- or TRPC1/5-transfected cells with the mock-transfected cells). (**b**–**f**) Scatter plots show mean values ± SEM and single values for the peak Fluo-4 AM intensity values of 6–8 cells per treatment for mock-transfected and TRPC1/4- or TRPC1/5-transfected CHO C6 cells (**b**,**e**), CHO 2A10 cells (**c**,**f**), and NCAM-lacking NCAM^neg^ CHO cells and in the absence (**b**–**f**) and presence (**e**,**f**) of SKF96365 (SKF) (* *p* < 0.05, ** *p* < 0.01 relative to mock-transfected (**b**) or vehicle-treated (**e**) cells; one-way ANOVA with Kruskal–Wallis post hoc test (**b**–**d**), Mann–Whitney test (**e**); ns, not significant). (**g**–**i**) Lysates of CHO 2A10 and CHO C6 cells were used for immunoprecipitation (IP) with TRPC1 antibody ACC-010 (**g**), TRPC4 antibody ACC-018 (**h**), TRPC5 antibody ACC-020 (**h**), and antibody GTX133217 against the C-terminus of NCAM (NCAM-CT) (**i**), and nonimmune control antibodies (Ig) (**g**–**i**) were used for immunoprecipitation. The lysates (input) and the immunoprecipitates were subjected to Western blot analysis with NCAM antibody GTX133217 (NCAM-CT) (**g**,**h**) or TRPC4 antibody ACC-018 (**i**).

To investigate whether PSA and/or NCAM regulate Ca^2+^ influx via TRPC1/4 and/or TRPC1/5 heteromers, CHO 2A10 cells expressing NCAM, but not PSA, and CHO C6 cells expressing PSA-NCAM were transfected with plasmids encoding TRPC1/4 or TRPC1/5 fusion proteins and subjected to Ca^2+^ imaging. To monitor the changes in intracellular Ca^2+^ levels, cells were loaded with the Ca^2+^ indicator Fluo-4 AM, and Fluo-4 AM signal intensity was recorded after treatment with or without the function-triggering NCAM antibody. NCAM antibody treatment of TRPC1/4- or TRPC1/5-expressing CHO C6 cells increased the Fluo-4 AM signal intensities, while no increase was observed in mock-transfected or non-triggered CHO C6 cells (Figure 8a,b). After antibody addition, a lag phase of 20–40 s was observed before the channels opened, after which Ca^2+^ signal intensities increased within 10–20 s. The channels then closed, and intensities declined to background levels within 10–20 s (Figure 8a). In contrast to TRPC1/4- or TRPC1/5-expressing CHO C6 cells, TRPC1/4- or TRPC1/5-expressing CHO 2A10 cells and TRPC1/4- or TRPC1/5-expressing NCAM-lacking NCAM^neg^ CHO cells did not show an increase in Fluo-4 AM signal intensities after NCAM antibody application (Figure 8c,d).

After pretreatment with the nonselective TRPC inhibitor SKF96365, the NCAM antibody-induced increase in Fluo-4 AM signal intensity was attenuated in TRPC1/4- and TRPC1/5-expressing CHO C6 cells, while the inhibitor had no effect in mock-transfected cells (Figure 8e). In mock-transfected and in TRPC1/4- or TRPC1/5-expressing CHO 2A10 cells, treatment with the NCAM antibody in the presence and absence of SKF96365 did not affect Fluo-4 AM signal intensities (Figure 8f). These results indicate that PSA is required for the NCAM-dependent regulation of the TRPC1/4- and TRPC1/5-mediated Ca^2+^ response. Of note, equal amounts of NCAM were immunoprecipitated with the TRPC antibodies, and equal amounts of TRPC4 were immunoprecipitated with an NCAM antibody from TRPC1/4- or TRPC1/5-expressing CHO 2A10 and CHO C6 cells (Figure 8g–i), indicating that the interaction of NCAM with these TRPCs does not depend on PSA.

**Figure 9:**
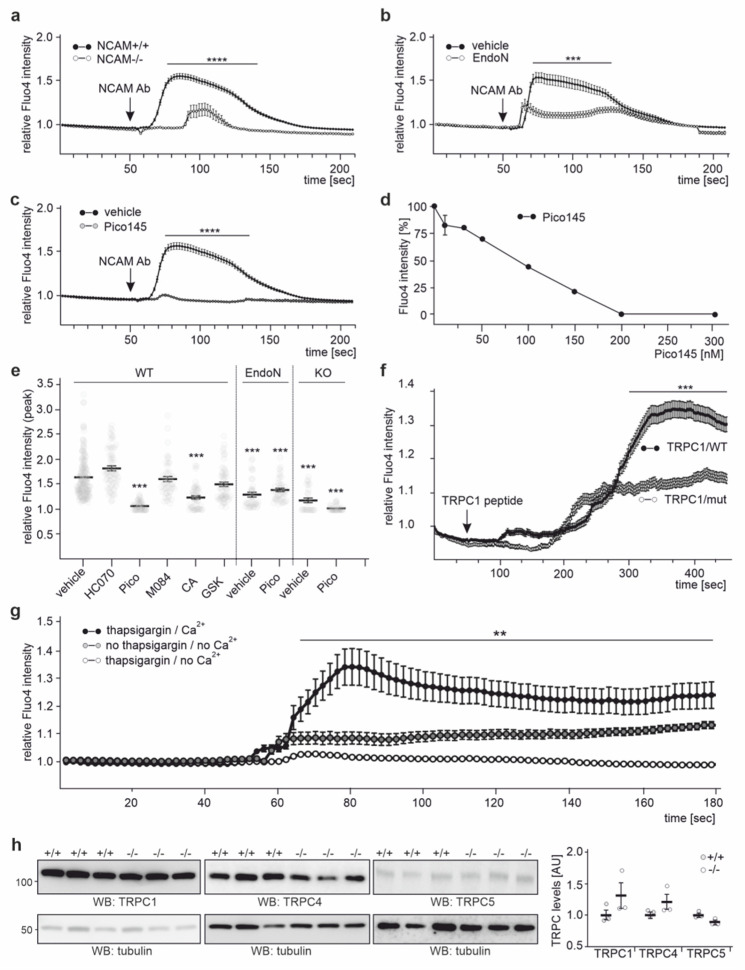
NCAM regulates the Ca^2+^ entry in cortical neurons via TRPC1, −4, and −5. Cortical neurons from wild-type (NCAM+/+) and NCAM-deficient (NCAM−/−) mice were treated without or with the PSA-degrading enzyme EndoN, loaded with Fluo-4 AM, preincubated with colominic acid/PSA (CA) or the TRPC inhibitors Pico145, HC-070, M084, or GSK-417651A, treated with or without function-triggering NCAM antibody, and subjected to Ca^2+^ imaging. Mean values ± SEM from 3 independent experiments analyzing 10 cells per treatment and experiment are shown for Ca^2+^ responses after treatment with function-triggering NCAM antibody (arrow; NCAM Ab) of wild-type and NCAM-deficient neurons (**a**), of non-treated and EndoN-treated wild-type neurons (**b**), and of wild-type neurons treated with or without Pico145 (**c**). (**d**) Pico145 reduces the NCAM-stimulated Ca^2+^ response in a concentration-dependent manner. Mean values ± SEM from 3 experiments analyzing 10 cells per experiment are shown. (**e**) Scatter plots show the peak Fluo-4 AM intensity values for wild-type neurons (WT), NCAM-deficient (KO) neurons, and EndoN-pretreated wild-type neurons (EndoN) after NCAM antibody treatment in the absence (vehicle) or presence of Pico145 (Pico), HC-070 (HC070), M084, GSK-417651A (GSK), or colominic acid/PSA (CA). Mean values ± SEM and single values from 3 experiments analyzing 10 cells per treatment and experiment are shown (** *p* < 0.01, *** *p* < 0.001, **** *p* < 0.0001 relative to values from vehicle-treated wild-type neurons; one-way ANOVA with Kruskal–Wallis post hoc test). (**f**) Representative time courses are shown for the Ca^2+^ responses after application of wild-type neurons with cell-penetrating unmutated tat-TRPC1/WT and mutated tat-TRPC1/mut peptides (arrow; TRPC1 peptides). (**g**) Cortical neurons were pretreated with thapsigargin in the absence of Ca^2+^, loaded with Fluo-4 AM, treated with NCAM antibody in the presence of Ca^2+^, and analyzed by Ca^2+^ imaging. Representative Ca^2+^ responses of thapsigargin-treated neurons after treatment with NCAM antibody (arrow; NCAM Ab) in the absence (thapsigargin/no Ca^2+^) or presence (thasigargin/Ca^2+^) of Ca^2+^ and of neurons not treated with thapsigargin and treated with NCAM antibody in the absence of Ca^2+^ (no thasigargin/no Ca^2+^) are shown. (**h**) Brain extracts from wild-type (+/+) and NCAM-deficient (−/−) mice were subjected to Western blot analysis with TRPC5 antibody 1C8, TRPC4 antibody ACC-018, TRPC1 antibody E-6, and α-tubulin antibody. TRPC1, −4, and −5 levels relative to α-tubulin levels were determined.

Next, we performed Ca^2+^ imaging with cortical neurons from wild-type and NCAM-deficient mice. After application of the function-triggering NCAM antibody, the Fluo-4 AM signal intensity was increased in wild-type neurons, while only a small increase was seen in neurons from NCAM-deficient mice (Figure 9a). In wild-type neurons, the channels opened after a lag phase of 10 s, and the signal intensities increased within 10 s before they slowly closed, and intensities decreased to background levels over 80–120 s (Figure 9a). Similar to NCAM-deficient neurons, only a slight increase in Fluo-4 AM signal intensity after NCAM antibody treatment was observed in wild-type neurons pretreated with EndoN [57] that degrades PSA (Figure 9b). Treatment of neurons with the TRPC1, −4, and −5 inhibitor Pico145 before the NCAM antibody application blocked the antibody-induced increase in Fluo-4 AM signal intensity (Figure 9c). Pico145 showed a concentration-dependent inhibition of the NCAM antibody-triggered Ca^2+^ response (Figure 9d). Pretreatment with the TRPC4 and −5 inhibitors HC-070 and M084 or the TRPC3, −6, and 7- inhibitor GSK-417651A did not affect the antibody-triggered increase in Fluo-4 AM signal intensity (Figure 9e). Interestingly, the application of colominic acid/PSA also inhibited the NCAM antibody-triggered increase in Fluo-4 AM signal intensity (Figure 9e), which we interpret to signify that soluble colominic acid/PSA saturates the binding sites on TRPCs so that they are not available for cell surface localized PSA-NCAM. Treatment of neurons with the cell-penetrating tat-TRPC1/WT peptide also triggered a long-lasting (>10 min) Ca^2+^ response, which started after a lag phase of 3–4 min and slowly declined after 7–8 min, while the mutated tat-TRPC1/mut peptide induced a considerably smaller Ca^2+^ response, which did not decline (Figure 9f).

Next, we analyzed whether NCAM-triggered Ca^2+^ responses via TRPCs are due to an influx of Ca^2+^. To test whether this influx depends on intracellular Ca^2+^ stores, stores were depleted by treatment with thapsigargin, which inhibits the retranslocation of Ca^2+^ from the cytoplasm to the ER. Pretreatment of neurons with thapsigargin in the absence of Ca^2+^ followed by treatment with the NCAM antibody in the presence of Ca^2+^ resulted in increased Fluo-4 AM signal intensities (Figure 9g). However, when neurons were pretreated with and without thapsigargin in the absence of Ca^2+^ and incubated with the NCAM antibody in the absence of Ca^2+^, Fluo-4 AM signal intensities were not increased (Figure 9g). These results show that the NCAM-dependent influx of extracellular Ca^2+^ via TRPCs does not depend on Ca^2+^ from intracellular stores, suggesting that NCAM-regulated TRPC1, −4, or −5 do not function as store-operated Ca^2+^ channels, but represent receptor-operated Ca^2+^ channels.

Of note, Western blot analysis showed equal levels of TRPC1, −4, and −5 in brain extracts from wild-type and NCAM-deficient mice (Figure 9h), indicating that the absence of NCAM does not alter TRPC1, −4, and −5 levels.

### 2.5. NCAM-Promoted Signal Transduction and Neurite Outgrowth Depend on TRPC1, −4, and −5

**Figure 10:**
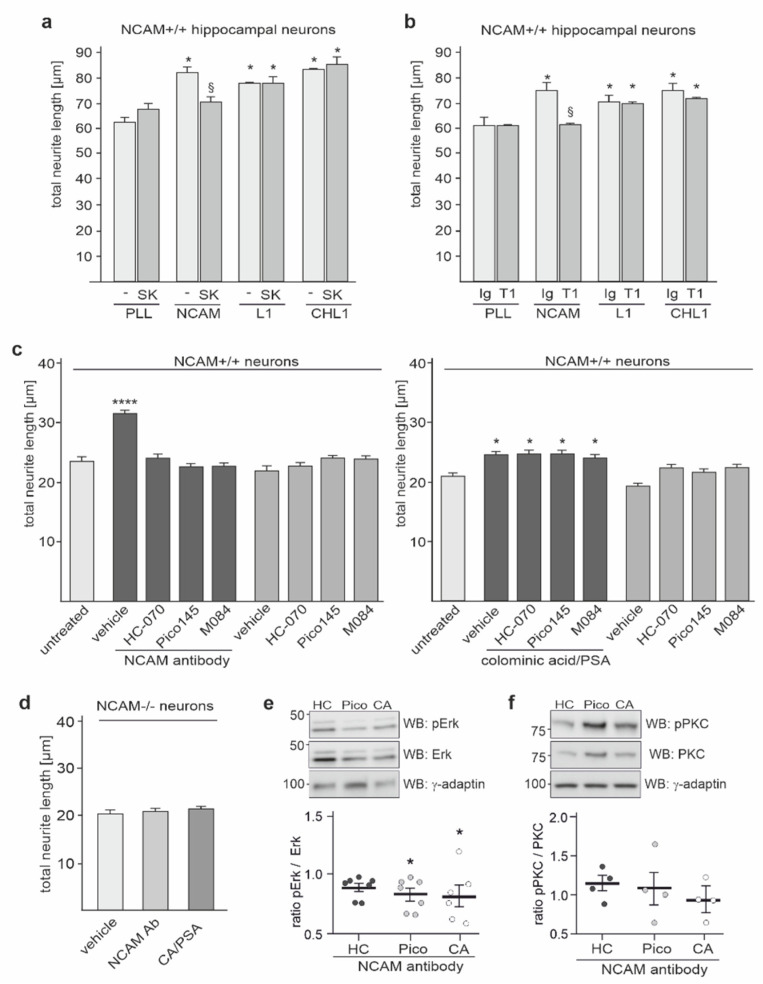
NCAM-mediated signal transduction and neurite outgrowth depend on TRPC1, −4, and −5. (**a**,**b**) Hippocampal neurons were maintained on PLL, NCAM-Fc (NCAM), L1-Fc (L1), or CHL1-Fc (CHL1) and treated without (−) (**a**) or with control antibody (Ig) (**b**), TRPC inhibitor SKF96365 (**a**), or function-blocking TRPC1 antibody T1E3 (**b**). Total neurite lengths per neuron were measured to determine neurite outgrowth. Mean values ± SEM from 3 independent experiments with duplicates are shown for neurite outgrowth relative to neurons without additives (* *p* < 0.01; one-way ANOVA with Dunn’s multiple comparison test) or relative to vehicle control (§ *p* < 0.01; one-way ANOVA with Dunn´s multiple comparison test). (**c**) Cortical neurons from wild-type (NCAM+/+) (**c**) and NCAM-deficient (NCAM−/−) (**d**) mice were treated with vehicle alone or together with NCAM antibody (NCAM Ab) or colominic acid/PSA (CA) in the absence (**c**,**d**) or presence of Pico145 (Pico), HC-070 (HC), or M084 (**c**). Total neurite lengths per neuron were measured to determine neurite outgrowth. Mean values ± SEM from 3 independent experiments with duplicates are shown for neurite outgrowth relative to neurons incubated without additives (* *p* < 0.05, **** *p* < 0.0001; one-way ANOVA with Kruskal–Wallis post hoc test). (**e**,**f**) Wild-type cortical neurons were treated with NCAM antibody in the absence or presence of HC-070 (HC) Pico145 (Pico) or colominic acid/PSA (CA) and subjected to Western blot analysis with antibodies against total and phosphorylated Erk1/2 and PKC and with a γ-adaptin antibody for loading control. Levels of total and phosphorylated Erk1/2 and PKC were determined by Western blot analysis. Mean values ± SEM are shown for the level of phosphorylated Erk1/2 (**e**) and PKC (**f**) relative to total Erk1/2 and PKC levels and relative to the values obtained for NCAM antibody-stimulated neurons in the absence of inhibitors (set to 100%) (* *p* < 0.05; one-way ANOVA with Kruskal–Wallis post hoc test).

Since NCAM is associated with TRPC1, −4, and −5, the question arises whether this association is of functional relevance. Of note, TRPC1, −4, and −5 as well as NCAM modulate cellular functions, such as neurite outgrowth [45,47,58,59]. To address the question whether the NCAM/TRPC interaction is involved in neurite outgrowth, we determined the neuritogenesis of hippocampal neurons on poly-l-lysine (PLL) and different neurite outgrowth-promoting substrates after incubation without or with the TRPC inhibitor SKF96365 or the inhibitory TRPC1 antibody T1E3. As substrates, we used recombinant NCAM-Fc, L1-Fc, or CHL1-Fc, which comprise the extracellular domains of the adhesion molecules in fusion with human Fc. On substrate-coated NCAM-Fc, neurite outgrowth was inhibited in the presence of the TRPC inhibitor SKF96365 or the inhibitory TRPC1 antibody, while neurite outgrowth on PLL alone and on L1-Fc and CHL1-Fc was not affected by these inhibitors (Figure 10a,b), suggesting that TRPCs assist only in NCAM-stimulated neurite outgrowth.

Next, we treated cortical neurons without or with the function-triggering NCAM antibody in the presence or absence of different TRPC inhibitors or colominic acid/PSA. Neurite outgrowth was enhanced by the NCAM antibody in the absence of inhibitors, while the NCAM antibody-triggered promotion of neurite outgrowth was reduced in the presence of the TRPC inhibitors Pico145, HC-070, and M084 (Figure 10c). However, neurite outgrowth promoted by colominic acid/PSA was not affected by the TRPC inhibitors (Figure 10c). NCAM antibody and colominic acid/PSA did not promote neurite outgrowth from NCAM-deficient neurons (Figure 10d). The results further support the notion that TRPCs are involved in cell surface-exposed NCAM-dependent and PSA-NCAM-dependent neurite outgrowth.

Since NCAM regulates the phosphorylation of PKC and Erk1/2 [13,15,60], we investigated whether the NCAM antibody-triggered phosphorylation of these signal molecules was affected by TRPC inhibitors. In parallel, the effect of colominic acid/PSA in the presence of the NCAM antibody was analyzed. Colominic acid/PSA as well as the TRPC1, −4, and −5 inhibitor Pico145, but not the TRPC4 and -5 inhibitor HC-070 reduced the NCAM antibody-induced Erk1/2 phosphorylation (Figure 10e), while neither the inhibitors nor soluble colominic acid/PSA affected the PKC phosphorylation (Figure 10f), indicating that the NCAM-triggered Erk1/2 phosphorylation depends on TRPC1, −4, and −5 and that cell surface-exposed PSA on NCAM and not soluble colominic acid/PSA is the functional trigger of these TRPCs and the Erk1/2 phosphorylation.

## 3. Discussion

By ELISA, we showed that not only the ICDs of NCAM140 and NCAM180, but also PSA bind to the N-terminal ICDs of TRPC1, −4, and −5, but not of other TRPCs. The results from pull-down and immunoprecipitation experiments verified that non-polysialylated and polysialylated NCAM140 and NCAM180 interact with TRPC1, −4, and −5 via their ICDs in brain tissue. Immunostaining and proximity ligation assays using cultured neurons reveal that these interactions take place in cerebellar, hippocampal, and cortical neurons. Colocalization of PSA and NCAM with TRPC1, −4, and −5 at the cell surface membrane of cortical neurons signifies interactions of PSA-NCAM with TRPCs at the cell surface of neurons. Moreover, live Ca^2+^ imaging in the absence and presence of TRPC inhibitors shows that the treatment of neurons with the NCAM antibody leads to the opening of TRPCs in the plasma membrane and to Ca^2+^ entry into the neurons via TRPC1, −4, and −5 hetero- or homomers. The NCAM-promoted opening of these Ca^2+^ channels does not depend on the release of Ca^2+^ from intracellular stores, but requires NCAM and PSA-NCAM, since the NCAM antibody-triggered Ca^2+^ influx is not observed in NCAM-deficient neurons. Thus, NCAM-associated TRPC1, −4, and −5 function as receptor-operated Ca^2+^ channels. The NCAM antibody-triggered Ca^2+^ influx is reduced in neurons after the removal of PSA from PSA-NCAM and is not observed in TRPC1/4- or TRPC1/5-expressing CHO cells that express NCAM, but not PSA. Thus, we conclude that the opening of NCAM-associated TRPCs requires PSA on NCAM. Of note, soluble colominic acid/PSA does not trigger a Ca^2+^ influx into neurons, but reduces the NCAM antibody-induced Ca^2+^ entry, most likely by saturating the PSA binding sites on TRPCs.

Based on these findings, we propose that the binding of the NCAM antibody to NCAM mimics the binding of an NCAM interaction partner and triggers a change of the NCAM conformation, which then could affect the binding of NCAM-ICD to the N-terminal ICDs of TRPCs and allow the PSA moiety of PSA-NCAM to bind to the N-terminal ICDs of TRPCs. It is conceivable that the binding sites for PSA and NCAM on the N-terminal ICDs of TRPCs overlap, since colominic acid/PSA and NCAM140-ICD bind to the TRPC1 peptide that contains the amphipathic helical structure-forming amino acids 267–278 of murine TRPC1. Under basal, non-stimulated conditions, NCAM140 and NCAM180 may bind via their ICDs to the N-terminal ICDs of TRPCs and may block the binding of PSA. An NCAM antibody-induced change in the conformations of NCAM or PSA-NCAM may then lead to the dissociation of the NCAM-ICD from its binding site on the N-terminal ICDs of TRPCs allowing the binding of PSA to its binding site in the N-terminal ICDs of TRPCs.

As was shown for MARCKS and PSA [54], cell surface-exposed PSA covalently bound to NCAM may then interact with the ICDs of TRPC-ICDs in the plane of the plasma membrane from opposite sites and may thereby trigger the transient opening of TRPCs and the Ca^2+^ influx, leading to increased cytoplasmic Ca^2+^ levels. Given that MARCKS binds to TRPC1 and PSA [54,55,56], it may be possible that MARCKS mediates the interaction of PSA with TRPC1 or contributes to this interaction between PSA and TRPC1.

Since stimulation by a function-triggering NCAM antibody results in the Ca^2+^-dependent binding of calmodulin to NCAM and to the Ca^2+^- and calmodulin-dependent proteolytic processing of NCAM and PSA-NCAM [16], it is tempting to speculate that the Ca^2+^-dependent binding of calmodulin to NCAM or a Ca^2+^-induced proteolytic cleavage of PSA-NCAM inactivates stimulation of TRPC by PSA-NCAM, thereby leading to the closing of TRPCs. The cell-penetrating TRPC1 peptide may compete with the binding of NCAM to its binding site in the N-terminal ICDs of TRPCs, and the binding of this peptide could mimic the binding of PSA attached to NCAM, resulting in long-lasting opening of TPRCs and in Ca^2+^ influx for more than 10 min. Since Pico145, but not HC-070, inhibits the NCAM antibody-triggered Ca^2+^ entry into neurons, the question arises as to the mechanisms of this difference. Although Pico145 and HC-070 are both xanthine derivatives and inhibit TRPC1, −4, and −5 channels with similar high potency [61,62,63], Pico145 and HC-070 bind to different sites on TRPC1, −4, and −5: Pico145 binds to lipid-binding sites thereby displacing phospholipids [64] that interact with the pore-forming helices of these TRPCs, while HC-070 binds to sites between adjacent TRPC molecules thereby replacing the glycerol group of DAG [65]. Thus, it is tempting to speculate that the phospholipids that are replaced by Pico145 are required for the binding of PSA to the ICD of TRPC1, −4, and −5 and, thus for the NCAM-induced opening of TRPCs, while the glycerol group of DAG that is replaced by HC-070 is not required for the binding of PSA to the ICD of these TRPCs and for the opening of these TRPCs.

The NCAM antibody-induced Ca^2+^ influx via TRPC1-, −4, and −5 results in an increase in cytoplasmic Ca^2+^ levels, which could trigger not only the activation of kinase- and phosphorylation-dependent signaling cascades [13,15,18,66], but also the Ca^2+^-dependent binding of calmodulin to NCAM and the Ca^2+^- and calmodulin-dependent nuclear import of NCAM and PSA-NCAM fragments [16,66,67,68]. Here, we show that the NCAM-dependent neurite outgrowth and Erk1/2 phosphorylation are inhibited by TRPC inhibitors and a function-blocking TRPC1 antibody as well by soluble colominic acid/PSA. Therefore, we conclude that NCAM-stimulated neurite outgrowth depends on the PSA-dependent opening of NCAM-associated TRPCs, which thereby regulate NCAM-dependent signal pathways and nuclear import of NCAM and PSA-NCAM fragments.

The interaction of PSA with MARCKS also contributes to the PSA-NCAM-dependent neurite outgrowth [54]. Of note, MARCKS interacts with TRPC1 and regulates the TRPC1-mediated Ca^2+^ entry: TRPC1-containing channels are closed when phosphatidylinositol 4,5-bisphosphate-carrying MARCKS binds to TRPC1, while the PKC phosphorylation of TRPC1, dissociation of MARCKS from TRPC1, release of phosphatidylinositol 4,5-bisphosphate from MARCKS, and binding of phosphatidylinositol 4,5-bisphosphate to TRPC1 induce the opening of TRPC1-containing channels in a calmodulin-dependent manner [55,56]. Based on these findings it is plausible that the interplay of TRPCs, MARCKS, and PSA-NCAM contributes to the regulation of neurite outgrowth.

From the results of our in vitro experiments, we infer that heteromeric TRPC1/4 and TRPC1/5 channels and homomeric TRPC4 and TRPC5 channels mediate NCAM-triggered Ca^2+^ entry in vivo. Thus, we propose that NCAM- and PSA-dependent Ca^2+^ entry via TRPC1, −4, and −5 regulates NCAM functions not only during development, such as neuritogenesis, synaptogenesis, and axon guidance, but also in the mature nervous system, such as synaptic plasticity and remodeling associated with learning, memory and behavior. In this context it is noteworthy that PSA, NCAM, and TRPC1, −4, and −5 regulate emotional behavior, in particular the formation and consolidation of fear memories and innate fear [68,69,70,71,72,73,74,75,76,77]. NCAM-deficient mice show anxiety-like behavior, which can be rescued by the transgenic expression of NCAM180 [69,70], while mice lacking NCAM in the forebrain showed impairments in innate and learned avoidance behaviors [71]. Several studies indicate that PSA-NCAM is involved in the consolidation of contextual memory. Mice deficient in polysialyltransferases ST8Sia-II and thus lacking PSA on NCAM showed not only higher exploration, but also reduced behavioral responses to Pavlovian fear conditioning [72], which is used as a behavioral paradigm for studying the formation of fear memories. In line with these findings is the observation that mice overexpressing a soluble fragment of extracellular NCAM are impaired in fear conditioning [73]. The exposure of wild-type mice to chronic unpredictable stress leads to enhanced NCAM expression in the amygdala, to enhanced fear conditioning, and to anxiety-like behavior [74,75]. Similarly, mice with NCAM deficiency in the forebrain show reduced NCAM levels in the amygdala and impaired auditory fear conditioning without exposure to chronic stress [74,75]. Enhanced PSA-NCAM expression in the dorsal hippocampus was observed after contextual fear conditioning, and removal of PSA by infusion of EndoN into the dorsal hippocampus reduces behavioral responses to contextual fear conditioning [76,77]. Similarly, after application to the dorsal hippocampus of wild-type mice, recombinant PSA-NCAM and colominic acid/PSA, but not recombinant NCAM, impaired the formation of contextual memory in fear conditioning paradigms [78]. The injection of recombinant PSA-NCAM into the hippocampus of NCAM-deficient mice could partially restore the impaired contextual memory in NCAM-deficient mice [78].

Behavioral abnormalities of mice abnormal in NCAM functions show some similarities to abnormalities of TRPC1-deficient mice, which are impaired in spatial working memory and fear conditioning [78] or TRPC4- and TRPC5-deficient mice characterized by reduced innate fear levels and increased exploratory behaviors [79,80]. Furthermore, the knockdown of TRPC4 in the lateral amygdala of wild-type mice also reduces innate fear levels and increases exploration [79]. The application of the TRPC4 and −5 inhibitor HC-070 attenuates the anxiogenic effect of a panic-inducing peptide in behavioral tests and ameliorates the increased fear memory induced by chronic social stress [80]. These findings suggest that emotional behavior may be regulated by the interaction between PSA-NCAM and TRPC1, −4, and −5 and by the PSA- and NCAM-dependent Ca^2+^ entry via TRPC1, -4, and -5. Moreover, these interactions and the Ca^2+^ influx may influence the interaction of the dopamine D2 receptor with NCAM180 and TRPC1, −4, or −5 [50,51] and thus could influence dopamine D2 receptor activity and dopamine-regulated emotional behavior including fear conditioning [81,82,83]. Impairments in the interplay among PSA-NCAM, dopamine D2 receptor, and TRPC1, −4, and −5 may lead to NCAM-and dopamine D2 receptor-associated psychiatric disorders, such as schizophrenia, bipolar disorder, depression, and anxiety disorder [6]. These interpretations should be investigated on the basis of molecular and cellular specificity in regard to different types of behavior in normal and diseased animals.

## 4. Materials and Methods

### 4.1. Animals and Cell Lines

Mice were bred and maintained at the animal facility of the Universitätsklinikum Hamburg-Eppendorf. NCAM-deficient mice [84] were provided by Harold Cremer (Developmental Biology Institute of Marseille, Marseille, France) and backcrossed onto the C57BL/6J background for more than eight generations. C57BL/6J or NCAM-deficient mice and their wild-type littermates of either sex were used in all animal experiments.

All animal experiments were conducted in accordance with the German and European Community laws on the protection of experimental animals and approved by the local authorities of the State of Hamburg (animal permit numbers N19/004_ZuchtNeuro, ORG 679 Morph and ORG 1022). The manuscript was prepared following the ARRIVE guidelines for animal research.

PSA-NCAM-expressing CHO C6 cells and PSA-negative NCAM-expressing CHO 2A10 cells have been described [68] and were a gift of Martina Mühlenhoff (Institut für Zelluläre Chemie, Medizinische Hochschule, Hannover, Germany). NCAM-lacking NCAM^neg^ CHO cells have been described [16].

### 4.2. Antibodies and Reagents

Monoclonal mouse antibody 5B8 against the ICDs of NCAM140 and NCAM180 was from the Developmental Studies Hybridoma Bank (Iowa City, IA, USA). Monoclonal rat antibody H28 against the extracellular NCAM domain, monoclonal rat antibody P61 recognizing the ICDs of NCAM140 and NCAM180, and monoclonal mouse antibody D3 directed against the sequence stretch encoded by the NCAM180-specific exon 18 have been described [13]. The mouse monoclonal PSA antibody 735 and EndoN were a gift of Rita Gerardy-Schahn (Zentrum Biochemie, Institut für Zelluläre Chemie, Medizinische Hochschule, Hannover) and have been described [54]. Polyclonal chicken (chNCAM-ECD) and guinea pig NCAM (gpNCAM-ECD) antibodies against the extracellular NCAM domain were produced by Pineda (Berlin, Germany) and described [68]. Polyclonal rabbit TRPC1 antibody H-105 (sc-20110) against amino acids 689–793 at the cytoplasmic C-terminus of human TRPC1; polyclonal rabbit TRPC4/5 antibody H-80 (sc-28760), which is raised against amino acids 1–80 at the cytoplasmic N-terminal domain of human TRPC5 and recognizes TRPC4 and −5; polyclonal rabbit TRPC3/6/7 antibody H-100 (sc-20111), which is raised against amino acids 1–100 at the cytoplasmic N-terminus of human TRPC3 and recognizes TRPC3, −6, and −7; monoclonal mouse TRPC1 antibody E-6 (sc-133076) against amino acids 689–793 at the cytoplasmic C-terminus of human TRPC1; goat polyclonal antibody C-18 (sc-34986) against the ICD of CHL1; α-tubulin antibody TU-02 (sc-8035); TRPC5 antibody 1C8 (sc-293259); and mouse monoclonal PKC antibody A-3 (sc-17769) were from Santa Cruz Biotechnology (Heidelberg, Germany). Monoclonal mouse TRPC4 antibody N77/15 against amino acids 930–947 at the cytoplasmic C-terminus of rat TRPC4 and TRPC5 antibody N67/15 against amino acids 827–845 at the cytoplasmic C-terminus of human TRPC5 were from NIH NeuroMab Facility (Davis, CA, USA; Hölzel Diagnostika, Cologne, Germany). Polyclonal rabbit TRPC1 antibody GTX54876 against amino acids 495–505 in the second extracellular domain of rat TRPC1 and polyclonal rabbit NCAM antibody GTX133217 against amino acids 730–1115 in the NCAM-ICD were from GeneTex (Irvine, CA, USA). The polyclonal rabbit anti-TRPC1 antibody ACC-010 against amino acids 557–571 in the intracellular loop of human TRPC1, rabbit anti-TRPC4 antibody ACC-018 against amino acids 943–958 in the C-terminal ICD of mouse TRPC1, the polyclonal rabbit anti-TRPC4 antibody ACC-119 against amino acids 458–469 in the second extracellular domain of rat TRPC4, and the polyclonal rabbit anti-TRPC5 antibody ACC-020 against amino acids 959–973 in the C-terminal ICD of human TRPC5 were from Alomone Labs (Jerusalem, Israel). The inhibitory antibody T1E3 against the third extracellular loop of TRPC1 [85] was a gift from Yao Xiaoqiang (The Chinese University of Hong Kong, School of Biomedical Science, Hong Kong, China). The chondroitin sulfate antibody CS-56 (Cat. No. C8035), colominic acid, and chondroitin sulfate were from Sigma-Aldrich (Deisenhofen, Germany). Antibodies against Erk1/2 (Cat. No. 4696) and phospho-Erk1/2 (Thr202/Tyr204) (Cat. No. 4370) and the phospho-PKC (pan; βII Ser660) antibody (Cat. No. 9371) were from Cell Signaling Technology Europe (Leiden, The Netherlands), and the γ-adaptin antibody (Cat. No. 610386) was from BD Biosciences (Heidelberg, Germany). All secondary antibodies were from Dianova (Hamburg, Germany) and primers from Metabion (Planegg, Germany). Recombinant NCAM-Fc, CHL1-Fc, and L1-Fc containing the extracellular portion of murine NCAM, CHL1, or L1 fused with the Fc fragment of human IgG and the recombinant His-tagged NCAM140-, NCAM180-, L1-, and CHL1-ICD have been described [16]. SKF96365 (CAS Number: 130495-35-1) was either from Sigma-Aldrich (Cat. No. S7809) or Biomol (Hamburg, Germany; Cat. No. Cay10009312). HC-070 (Cat. No. HY-112302; CAS Number: 1628291-95-1) and Pico145 (Cat. No HY-101507; Cas Number: 1628287-16-0) were from Hölzel Diagnostika; M084 (Cat. No. 5807; CAS Number: 1992047-63-8) was from Bio-Techne (Minneapolis, MN, USA), and GSK-417651A (Cat. No. FBM-10-1434; CAS number: 736945-96-3) was from Biozol (Eching, Germany). Vectors encoding human TRPC1 or mouse TRPC4 or −5 were kindly provided by Markus Delling (Department of Cardiology, Children´s Hospital, Boston, MA, USA). The TRPC1 peptide (RNDYEELARQCKMFAKDLLAQARNSRELE) as well as the cell-penetrating peptides tat-TRPC1/WT (YGRKKRRQRRR-RNDYEELARQCKMFAKDLLAQARNSRELE) and tat-TRPC1/mut (YGRKKRRQRRR-QNDYEELAQQCQMFAQDLLAQAQNSQELE) were from Schafer-N (Copenhagen, Denmark).

### 4.3. Cloning of TRPC1/4 and TRPC1/5 Heteromers

cDNAs encoding for TRPC1/4 and TRPC1/5 fusion proteins were amplified by PCR using pcDNA3-TRPC1, pcDNA3-TRPC4, and pcDNA3-TRPC5 plasmids as templates and the following primer combinations: TRPC1_D (5′-ggctctgactgaccgcgtta atg atg gcg gcc ctg tac c-3′) and TRPC1_B (5′-a ctg agc cat att tct tgg ata aaa cat agc-3′), TRPC1_D and TRPC1_C (5′-g ctg ggc cat att tct tgg ata aaa cat ag-3′), TRPC4_A (5′-t cca aga aat atg gct cag ttc tat tac aaa aga a-3′) and TRPC4_C (5′-ctgtgctggcggccggc tca caa tct tgt ggt cac ata atc t-3′), or TRPC5_A (5′-t cca aga aat atg gcc cag ctg tac tac aag-3′) and TRPC5_C (5′-ccactgtgctggcggccggc tta gag ccg agt tgt aac tt gtt c-3′). The PCR products TRPC1_D/TRPC1_B and TRPC4_A/TRPC4_C as well as the TRPC1_D/TRPC1_C and TRPC5_A/TRPC_C were cloned into the linearized expression vector pCAGIG using In-Fusion^®^HD Cloning Kit (Takara Bio Europe, Saint-Germain-en-Laye, France). For linearization, pCAGIG was subjected to PCR with the primers pCAGIG_A (5′-gccggccgccagcacagtg-3′) and pCAGIG_B (5′-taacgcggtcagtcagagcc-3′).

### 4.4. Cloning and Expression of the N- and C-Terminal ICDs of TRPC1, 4, and 5

cDNAs coding for the N-terminal ICDs of human TRPC1 (1N; amino acids 1–386), mouse TRPC4 (4N; amino acids 1–329) and TRPC5 (5N; amino acids 1–330) or coding for the C-terminal ICD of human TRPC1 (1C; amino acids 638–793) were amplified using pcDNA3 plasmids with TRPC1, −4, and −5 cDNAs (a gift of Markus Delling, Cardiology, Children´s Hospital, Boston, MA, USA) and the following primers: 1N fw (5′-aaa ccc ggg atg atg gcg gcc ctg tac ccg a-3′), 1N rev (5′-ttt ccc ggg ttt cat aaa agg tgt gtg aat gat tct-3′), 1C fw (5′-aaa ccc ggg acc aaa ctg ctg gtg gca atg ct-3′), 1C rev (5′-ttt ccc ggg att tct tgg ata aaa cat agc ata ttt ag-3′), 4N fw (5′-aaa ccc ggg atg gct cag ttc tat tac aaa aga aat-3′), 4N rev (5′-ttt ccc ggg ctt cac cgc cca gtg tct tct c-3′), 5N fw (5′-aaa ccc ggg atg gcc caa ctg tac tac aaa aag g-3′), and 5N rev (5′-ttt ccc ggg ctt gac tac cca gtg ttt ccg cc-3′). The PCR products were cloned into the pGEX vector coding for a N-terminal GST-tag. Production and purification of recombinant protein have been described [16,51].

### 4.5. Cloning and Expression of His-Tagged NCAM140-ICD with Deletions of the N-Terminal, Middle, or C-Terminal Part

For deletion of N-terminal amino acids 1–44 or of the C-terminal amino acids 76–120, cDNA encoding 120 amino acids of the rat NCAM140-ICD and the primers 5′-agcgggatcc gag tct aaa gaa ccc att gta-3′ containing a KpnI site and 5′-ttttggatcc tca tgc ttt gct ctc att ctc-3′ containing a BamHI site or 5′gataggatcc gac atc acc tgc tac ttc ctg-3′ and 5′-gataggatcctca tgt ggt ctc gtt ggg ctc-3′ containing KpnI sites were used for PCR. For deletion of the middle amino acids 29–66, the primers NCAM-inner (5′-c atg tgc atc gct gtt aac ctg tgc ggc aaa gcg ggg ccc gga aag cac aca gag ccc aac gag ac-3′) and NCAM-HindIII rev (5′-tcgtaagctt tca tgc ttt gct ctc att ctc-3′; containing a HindIII site) were first used for PCR with NCAM140-ICD cDNA, and then the resulting PCR product was used for amplification with the primers NCAM-outer (5′-atg gac atc acc tgc tac ttc ctg aac aag tgt ggc ctg ctc atg tgc atc gct gtt aac ctg t-3′) and NCAM-HindIII rev. Finally, the resulting PCR product was amplified using the primer 5′-gataggtacc gac atc acc tgc tac ttc ctg-3′ containing a KpnI site and NCAM-HindIII rev. The restriction sites were used to insert the cDNA constructs into the expression vector pQE30, and the resulting vectors were used for transformation and protein expression in *E. coli* M15.

### 4.6. Immunoprecipitation, Pull-Down, and Western Blot Analysis

Brains from adult mice were homogenized at 4 °C in lysis buffer (50 mM Tris-HCl, pH 7.5, 150 mM NaCl, 1% Nonidet P-40, and 1 mM Na_4_P2O_4_) containing protease inhibitor cocktail (Complete, EDTA free; Roche) and centrifuged at 1000× *g* for 5 min. The resulting supernatant was taken as detergent extract for immunoprecipitation and pull-down experiments [16,51].

For preparation of cell lysates, CHO cells were washed twice with cold phosphate-buffered saline (PBS), incubated for 15 min with gentle shaking in ice-cold iMAC buffer (20 mM HEPES, pH 7.2, 100 mM potassium acetate, 40 mM KCl, 5 mM EGTA, 5 mM MgCl_2_, and phosphatase and protease inhibitor cocktails (PhosSTOP EASYpack and cOmplete EDTA-Free Protease Inhibitor Cocktail (Sigma-Aldrich)) containing 1% Triton X-100 (iMAC–Triton buffer). Cell lysates were harvested using a cell scraper.

For preparation of membrane-enriched fractions, the brain homogenates or CHO cell lysates were centrifuged for 10 min at 1000× *g* and 4 °C. The supernatants were then centrifuged for 10 min at 10,000× *g* and 4 °C. The resulting pellets were resuspended in iMAC–Triton buffer and used as membrane-enriched fractions.

In pull-down experiments, 5 µg His-tagged NCAM140-ICD or GST-tagged N-terminal TRPC1-ICD, TRPC4-ICD, and TRPC5-ICD was incubated with Ni–NTA agarose beads or glutathione–agarose beads, respectively, for 1 h at 4 °C under gentle shaking. Beads were washed two times with PBS containing 1% Triton X-100 (PBS–Triton) for 10 min and gentle shaking and incubated with brain homogenates for 2 h at 4 °C under gentle shaking. Finally, beads were washed 2 times with iMAC–Triton buffer for 10 min and once with iMAC buffer for 10 min. Beads were resuspended in sample buffer and boiled at 95 °C for 5 min.

For coimmunoprecipitation, 3–5 μg of primary antibodies was incubated for 1 h at 4 °C and gentle shaking with Protein G or Protein A Dynabeads^TM^ prewashed in PBS–Triton. After two washes with PBS–Triton and one wash with iMAC–Triton buffer, the beads were incubated with the detergent brain extract or the CHO cell lysates for 2 h at 4 °C under gentle shaking. Finally, beads were washed 2 times with iMAC–Triton buffer, washed 2 times with iMAC buffer, resuspended in sample buffer and boiled at 95 °C for 5 min.

Western blot analysis was performed as described [16]. Briefly, proteins separated by SDS-PAGE were transferred to nitrocellulose membranes. The membranes were incubated for 1 h at room temperature in 5% skim milk powder in Tris-buffered saline (TBS) containing 0.01% Tween-20 (TBS-T), washed 3 times with TBS-T, and incubated overnight at 4 °C with the primary antibody diluted with 5% skim milk powder in TBS-T. After 3 washes for 10 min in TBS-T, the membranes were incubated for 1 h with horseradish peroxidase-coupled secondary antibody and washed 3 times with TBS-T for 10 min. For visualization of bands, ECL select or ECL prime reagents (Thermo Fisher Scientific, Darmstadt, Germany) and the LAS4000 Mini (GE Healthcare, Freiburg, Germany) were used.

### 4.7. ELISA

Recombinant proteins or peptides were diluted to 10 µg/mL in PBS, and 25 µL of the dilutions per well was used for coating at 4°C overnight in 384-well microtiter plates with high-binding surface (Corning, Tewksbury, MA, USA). The wells were then incubated with blocking solution (2% essentially fatty acid-free BSA (Sigma-Aldrich) in PBS at room temperature for 1.5 h, washed with PBS for 30 sec, and incubated at room temperature for 1.5 h with different concentrations of recombinant proteins in the presence of different concentrations of peptides. After washing 3 times with PBST (PBS with 0.05 % Tween 20) for 30 s, primary antibodies (diluted 1:300 in PBS) were applied for 1 h. The ELISA plate was washed 3 times for 5 min with PBST and incubated for 1 h with horseradish peroxidase-conjugated secondary antibody (diluted 1:1000 in PBS). The plate was rinsed again 3 times with PBST for 5 min. As horseradish peroxidase substrate, 25 µL ortho-phenylenediamine dihydrochloride (Thermo Fisher Scientific; 1 mg/mL) was added per well, and plates were incubated for 0.5–5 min. The color reaction was terminated with 2.4 M sulfuric acid, and the absorption was measured at 492 nm using the μQuantTM microplate spectrophotometer (Bio-Tek Instruments, Bad Friedrichshall, Germany).

### 4.8. Primary Culture of Mouse Cerebellar, Cortical, and Hippocampal Neurons

Hippocampal neurons were isolated from mouse embryos at embryonic day 15.5 as described [51] and maintained in BrainPhys Neuronal medium supplemented with 1% L-glutamine, 1% Pen/Strep, and 2% NeuroCult SMI Neuronal Supplement (STEMCELL Technologies, Cologne, Germany; Cat. No.: 05792). The preparation of cortical neurons from 16- to 18-day-old mouse embryos and the preparation of cerebellar neurons from 6- to 8-day-old mice have been described [86]. Cortical neurons were maintained in culture medium containing Neurobasal medium, 2 mM L-glutamine, 1% Pen/Strep, and B27 supplement, and cerebellar neurons were maintained in Neurobasal A medium (Thermo Fisher Scientific) supplemented with 2 mM L-glutamine (Thermo Fisher Scientific), 0.1 mg/mL streptomycin and 10 U/mL penicillin (Thermo Fisher Scientific), 1 mg/mL BSA (Sigma-Aldrich), 10 μg/mL insulin (Sigma-Aldrich), 4 nM L-thyroxine (Sigma-Aldrich), 100 μg/mL transferrin (Merck, Darmstadt, Germany), 30 nM sodium selenite (Sigma-Aldrich), 1 mM sodium pyruvate, and B27 supplement containing no or 5% fetal horse serum. Hippocampal and cortical cells were seeded at a concentration of 5 × 10^4^ cells/well for proximity ligation assay, TIRF analysis, and immunostainings; 2 × 10^6^ cells/well for Western blot analysis; 2.5 × 10^5^ for Ca^2+^ imaging; and 2.5 × 10^4^ cells/well for neurite outgrowth, while 5 × 10^5^ cerebellar cells/well were seeded for immunostainings. Cells were cultured for 5 to 7 days at 37 °C in 5% CO_2_ atmosphere and 90% humidity.

### 4.9. Culture of CHO Cells

CHO cells were maintained DMEM/F12 (1:1) medium supplemented with 2 mM L-glutamine, 1% Pen/Strep, 5% fetal calf serum, and 1 mM sodium pyruvate. For immunoprecipitation experiments or Ca^2+^ imaging, 3 × 10^6^ or 1.5 × 10^6^ cells/well were seeded.

### 4.10. Transfection of Cortical Cells and CHO Cells

For transfection of cortical neurons, cells were maintained in culture for 5 days, and 2–3 μg of plasmid was added together with LipofectamineTM 2000 (Thermo Fisher Scientific) according to the manufacturer’s instructions. After 1 h, the cells were washed 2 times with HEPES buffer (10 mM HEPES, pH 7.5, 135 mM NaCl, 5 mM KCl, 15 mM glucose, 2 mM MgCl_2_, and 2 mM CaCl_2_) and maintained in culture medium. Finally, cells were kept in culture for 2 days.

For transfection of CHO cells, cells were kept for 1 day in culture in a 6-well plate before transfection, and 3 μg of plasmid was added to the cells together with 3 μL of PlusTM Reagent and 9 μL of LipofectamineTM LTX (Thermo Fisher Scientific) following the manufacturer’s protocol. After 6 h, cells were washed 2 times with HEPES buffer, and maintenance medium was again added. Finally, cells were kept in culture for 1 day.

### 4.11. Immunocytochemistry and Analysis of Colocalization

Immunostaining after fixation has been described [16]. Briefly, cells seeded on glass coverslips were fixed in 4% formaldehyde for 15 min at room temperature. After three washes with PBS for 5 min, cells were incubated for 1 h with 5% donkey serum in PBS. For permeabilization, cells were incubated with 0.25% Triton X-100 and 5% donkey serum in PBS. Primary antibodies were diluted in PBS and incubated overnight at 4 °C. After 3 washes with PBS for 5 min, cells were incubated for 1 h at room temperature with secondary antibodies and 5 µg/mL 4′,6-diamidino-2-phenylindole (DAPI) in PBS and washed 3 times for 5 min in PBS. Coverslips were then mounted using mounting medium (Shandon Immu-Mount^TM^, Thermo Fisher Scientific), and images were taken with a confocal microscope (Olympus FV1000; Olympus, Hamburg, Germany) using a 60× objective. For analysis of colocalization, Pearson’s analysis of images was performed using the Coloc 2 plugin of the ImageJ software to calculate how many pixels of two different stainings overlap with each other. For every dual-channel image, the pixel distribution diagram was used to calculate the Pearson’s coefficient, which gives information about the linear correlation between pixel intensity in the green and red images and measures the pixel-by-pixel covariance in the signal levels of two images, as it subtracts the mean intensity from each pixel’s intensity value [87,88]. The Pearson coefficient is independent of signal levels and signal offset (background), and the value of the coefficient ranges between 1 for perfect correlation, 0 for no correlation, and −1 for total negative correlation.

### 4.12. Proximity Ligation Assay

Proximity ligation assay was performed using the Duolink^®^ Proximity Ligation Assay (Sigma-Aldrich) according to the manufacturer’s instructions. Cells were fixed in 4% formaldehyde and were either permeabilized for 15 min using 0.25% Triton X-100 in PBS before blocking or were directly incubated in Duolink blocking solution for 30 min. The primary antibodies were diluted in Duolink AB Diluent and incubated overnight at 4 °C. For confocal microscopy, coverslips were finally incubated with 5 µg/mL DAPI for 5 min and washed 2 times with PBS for 10 min and mounted. Images were taken with a confocal microscope (Olympus FV1000) using a 60× objective. For spinning disk microscopy, cells were kept in HEPES buffer, and images were taken at a spinning disk microscope (Nikon Eclipse Ti, Nikon, Düsseldorf, Germany).

### 4.13. Total Internal Reflection Fluorescence (TIRF) Microscopy

For TIRF microscopy, cortical cells were first transfected with tagBFP2-CAAX (plasmid #116856; Addgene, Watertown, MA, USA) [89] and pMH4-SYN-EGFP-ER (plasmid #22285; Addgene) [90]. The plasmid tagBFP2-CAAX encodes a fusion protein comprising blue fluorescence protein (BFP) and the CAAX motif-containing amino acids 172–184 of the proto-oncogene H-Ras, and the plasmid pMH4-SYN-EGFP-ER encodes a fusion protein comprising enhanced green fluorescence protein (EGFP), the ER-targeting sequence of calreticulin, and the ER retention sequence KDEL.

Then, cells were fixed in 4% formaldehyde and subjected to proximity ligation assay. Finally, cells were kept in HEPES buffer, and images were taken with a Nikon Eclipse Ti spinning disk microscope equipped with a Nikon CFI Apo TIRF 100× 1.49 N.A. oil objective using 405 nm, 488 nm, and 561 nm lasers. Images were recorded using Visitron Systems software (Visitron Systems, Puchheim, Germany), and numbers of red dots were quantified using ImageJ.

### 4.14. Analysis of Neurite Outgrowth

Neurite lengths were determined as described [52]. Briefly, cortical and hippocampal cells were seeded on PLL-coated 48-well plates. After 30 min, the cells were treated with 200 nM HC-070, 200 nM Pico145, 50 μM M084, 10 μM SKF-96365, or 10 μg/mL colominic acid for 15 min. Dimethyl sulfoxide (DMSO) was used to dissolve the TRPC inhibitors and served as vehicle control at a final concentration of 0.1%. Function-triggering guinea pig NCAM antibody was added to the cells. After 30 h, cells were fixed in 2.5% glutaraldehyde for 30–60 min at 37 °C, washed 3 times with H_2_O, and stained with 1% toluidine blue and 1% methylene blue in 1% sodium tetraborate for 1 h at room temperature. Neurite outgrowth was analyzed by measuring the total length of neurites in an Axiovert microscope with the AxioVision 4.6 imaging system (Zeiss, Oberkochen, Germany).

### 4.15. Ca^2+^ Imaging of Cortical Neurons and CHO Cells

Cortical neurons were maintained in culture for 4–5 days. For EndoN pretreatment, neurons were incubated without (vehicle) or with 5 μg/mL EndoN for 1 h. For pretreatment with thapsigargin, cortical neurons were washed once with prewarmed HEPES buffer and incubated for 3 h with 1 μM thapsigargin in HEPES buffer without CaCl_2_.

For Fluo-4 loading, cortical neurons or CHO cells were maintained for 30 min in pre-warmed HEPES buffer with 0.02% Pluronic F-127 and 2.5 μM or 5 μM Fluo-4 AM, respectively. Cortical cells were washed 2 times with Hank’s Balanced Salt Solution and incubated for 20 min with prewarmed HEPES buffer without or with 200 nM HC-070, 200 nM Pico145, 200 nM GSK-417651A, 50 μM M084, or 10 μg/mL colominic acid. DMSO at 0.1% served as vehicle control. CHO cells were washed 2 times with Hank’s Balanced Salt Solution and incubated for 20 min with prewarmed HEPES buffer with DMSO at 0.1% (vehicle) or with 10 μM SKF-96365. Images were taken every second using a spinning disk confocal microscope (Nikon Eclipse Ti), a 20× objective and the Visitron Systems software. The function-triggering guinea pig NCAM antibody was applied after 50 sec of recording, and the total video recording time was 5 min. Finally, the intensity of the Fluo-4 AM signal was analyzed using ImageJ software.

### 4.16. Statistical Analysis

Analyses were performed using the GraphPad Prism 8 software. The types of tests are indicated in the legends. *p*-values of <0.05, <0.01, <0.001, and <0.0001 were accepted as a significant difference and indicated by *, **, ***, and ****.

## Data Availability

Not applicable.
